# Efficient Work

**Published:** 1891-06

**Authors:** 


EFFICIENT WORK.—The JOURNAL has authority for the statement that Texas is, to-day, almost entirely free from small-pox, there not being, all told, within its vast domain, one hundred cases.

When we reflect that six months ago there were over two thousand cases in the State, and it looked as if the pest had come to stay, converting Texas into a second Mexico,—when consternation was spread abroad, and people were afraid to come to Texas,—the present showing is most gratifying. It can mean but one thing,—efficient management. Much credit is due the State Health Officer for his prompt, energetic and intelligent action in the premises, and for his judicious choice of sanitary officers; and credit is due them also. The State Health Officer is the author of the quarantine bill now on the books, whereby the whole machinery for the carrying out of internal quarantine has been put in operation. This bill was published in the JOURNAL in March. It creates a health officer in each county and city, subordinate to the State Health Officer, and the above excellent showing is the first fruits of this judicious legislation.

